# Trends in the diversity of mortality causes and age-standardised mortality rates among subpopulations within Scotland, 2001–2019

**DOI:** 10.1016/j.ssmph.2022.101192

**Published:** 2022-08-13

**Authors:** Ciaran McMonagle, Denise Brown, Richard Reeve, Rebecca Mancy

**Affiliations:** a MRC/CSO Social and Public Health Sciences Unit, University of Glasgow, G3 7HR, UK; b Boyd Orr Centre for Population and Ecosystem Health, University of Glasgow, Glasgow, UK; c School of Biodiversity, One Health and Veterinary Medicine, College of Medical, Veterinary and Life Sciences, University of Glasgow, Glasgow, G12 8QQ, UK

**Keywords:** Causes of mortality, Diversification, Inequality, Deprivation, Scotland

## Abstract

Previous research has demonstrated increasing diversity in causes of mortality among high-income nations in recent decades, associated with improvements in health and increasing life expectancies. Health outcomes are known to vary widely between communities within these countries and inequalities between sexes and other subpopulations are key in understanding the health of populations. Despite this, little is known about variation in the diversity of mortality causes between these subpopulations. Diversification in mortality causes indicates an increase in the pool of potential causes of mortality an individual is likely to face. This poses challenges for the public health and medical sectors by increasing diagnostic uncertainty and broadening the range of causes to be addressed by public health and medical interventions. Here we examine trends over time in the diversity in causes of mortality in Scotland by sex and area-level deprivation, also examining deaths among those younger than 75 years and those 75 years and older separately. We find that diversity in causes of mortality has increased across subpopulations; that it has risen more quickly in men than women; that the rate of increase has been similar across age categories; and that there is no clear ranking in the trends by deprivation quintile, despite slower improvements in mortality rates among the most deprived. Increasing diversity in mortality causes suggests that a greater public health focus on reducing death rates from a broader range of causes is likely to be required, and this may be especially important for men who face a faster rate of diversification.

## Highlights

• Causes of mortality in Scotland became more diverse in the period 2001–2019.
• Diversification indicates deaths are more likely to be attributed to uncommon cause.
• Mortality causes have diversified more quickly in men than women.
• The relationship between diversity of causes of mortality and deprivation is unclear.
• Increased focus on reducing mortality from a broader range of causes is required.

## 1 Introduction

From the latter half of the 20th century onwards, high-income, developed countries have seen a transition in the profile of mortality causes affecting their populations (Ursache et al., 2021). This transition has been wide-ranging, but most notably has seen reductions in the rates of mortality from diseases of the cardiovascular system and malignant neoplasms (cancers), previously the two most common causes of mortality in these countries (Bongaarts, 2014). These reductions have been achieved through a combination of improved medical therapies and reductions in major risk factors (Bhatnagar et al., 2016; Capewell & Graham, 2010; Ford et al., 2007; Lopez & Adair, 2019; Marshall et al., 2016) and contributed in large part to the fall in all-cause mortality in these countries. Over this time, the rate of death associated with diseases of the cardiovascular system fell more quickly than that of cancers. Due to this, cancers have succeeded diseases of the cardiovascular system as the leading mortality cause in many countries including the UK and much of Western Europe (Brown et al., 2019; Pereira et al., 2012; Wilson et al., 2017). The associated reductions in mortality rates have led to increases in life expectancies, although in recent years these increases have stalled (Fenton et al., 2019; Hiam et al., 2018; Leon et al., 2019; Ramsay et al., 2020). In turn, many countries have seen a rise in the prevalence of degenerative diseases and other age-related conditions (Murphy, 2021).

As rates of mortality associated with the most prevalent causes of death have reduced, there has been a prevailing trend towards a more even distribution of deaths across mortality causes (Bergeron-Boucher et al., 2017; Gregg et al., 2018). In other words, the causes of mortality within the population have diversified. If one were to pick a death at random from the population, the likelihood of it being caused by cancer or cardiovascular diseases has fallen. Conversely, the chance of that death being caused by another mortality cause – many of which have become proportionally more prevalent – has increased. We can examine this variation in the distribution of mortality causes by applying measures of diversity to mortality data. These measures allow us to compare how mortality is distributed across causes, and to compare between populations and across time.

Diversification in the causes of mortality within a population brings changes to the challenges faced by the healthcare system. As deaths become more evenly distributed between causes of mortality, causes which may have been rare in the past make up a proportionally larger number of deaths. This has the potential to increase the diagnostic uncertainty associated with each patient (Bergeron-Boucher et al., 2020; Bhise et al., 2018). At the same time, current economies of scale in the prevention and treatment of common causes of mortality become progressively less effective as their benefactors make up a smaller proportion of the population. As causes of death within the population diversify, there is a larger pool of causes that are likely to cause morbidity as well as mortality. This also increases the burden on public health systems and officials as resources must be directed to an increasing range of significant causes of mortality.

Diversity in causes of mortality has been previously compared across countries in a limited number of studies, although variation within countries has yet to be studied (Bergeron-Boucher et al., 2020; Izsak, 1993). Bergeron-Boucher et al. (2020) find that across a range of high-income countries including the United Kingdom, the diversity of mortality causes increased from the 1990s to the 2010s; they suggest that increasing life expectancies are a key factor in this diversification of deaths because age is an important risk factor in many diseases.

Despite the importance of inequalities in health within countries (Wyper et al., 2019), previously comparisons of diversity in mortality causes have only been made between sexes. Key subpopulations between which inequalities in health outcomes are considered to be important include sexes and area-level economic deprivation; within these subpopulations age at death is also a key consideration. Examining trends in the diversity of mortality causes in these groups provides aninsight into disparities in health between these subpopulations. Regarding sex differences, women have previously been noted to face a more diverse set of mortality causes (Bergeron-Boucher et al., 2020); however, differences in the trends in diversification between sexes have not been examined. The aging of populations has previously been suggested as a driver of diversification, but diversity in mortality cause among subpopulations dying at different ages has not been explicitly studied Economic deprivation has not previously been examined as a driver of variation in diversity of mortality causes, despite the importance placed on inequalities in all-cause mortality and cause-specific mortality between groups (Brown et al., 2019). In summary, despite acknowledged differences in health outcomes between subpopulations by sex, age, and deprivation, comparisons of diversification in causes of mortality between these subpopulations are missing from the literature.

Here we study the diversity of mortality causes within Scotland, distinguishing between sexes, by premature mortality and mortality at older ages, and by area-level economic deprivation. We choose to focus our study on Scotland because it faces higher mortality rates and wider inequalities in health outcomes than comparable countries and is therefore a country for which the public health imperative is especially clear (Mccartney et al., 2012). High inequalities in health in Scotland further mean we may expect greater variation in the distribution of causes of mortality between subpopulations than in more equitable countries. Increases in the diversity of causes of mortality have previously been observed at the level of the United Kingdom (Bergeron-Boucher et al., 2020). We can expect similar trends in Scotland; however, health outcomes have improved more slowly than in other regions of the UK and what effect this may have on the diversity in causes across Scotland and in subpopulations is unknown.

## 2 Methods

### 2.1 Mortality

We use individual-level mortality records from National Records of Scotland (NRS) vital events data for the years 2001 to 2019, totalling 1,059,112 deaths. The highest yearly death count is found in 2017 at 58,285 and the lowest in 2011 at 53,469, with a mean of 55,720 deaths per year. A small number of records were incomplete or there were issues with geographic identifiers, and 174 records (deaths) were thus excluded from analysis. The remaining records were used to calculate diversity in causes of mortality and (alongside NRS population data) age-standardised mortality rates, as described below.

Individual causes of mortality were classified at the level of International Statistical Classification of Diseases (10th revision) (ICD-10) three-character codes, extracted from the NRS mortality data (World Health Organization, 2004). Three-character codes are the primary breakdown of causes of mortality within the ICD-10 system. Here, each mortality record was assigned a three-character code and each code present in the data was treated as an individual cause of mortality. Supplementary Fig. 1 shows the number of ICD-10 three-character code causes of mortality recorded in each year; this is presented in the form of normalised alpha diversity at q = 0, which is equivalent to (and thus can be read as) a count of the total number of causes.

### 2.2 Population

Mid-year small area population estimates for the years 2001–2019 were obtained from National Records of Scotland (NRS) (National Records of Scotland, 2020). The total population of Scotland was 5,064,200 in 2001, rising to 5,463,300 in 2019.

### 2.3 Area-level deprivation

Population-weighted income deprivation quintiles for Scotland were calculated from the income domain of the Scottish Index of Multiple Deprivation (SIMD). SIMD is a widely used relative measure of area-level deprivation computed by the Scottish Government. The income domain of the SIMD is used here because the full index takes into consideration aspects of deprivation that directly measure aspects of the health of populations. SIMD 2006 was used for the years 2001–2009, with SIMD 2016 used for the years 2010–2019. We use datazones as the areal unit. Datazones are small area geographies used by the Scottish Government for a range of statistical outputs with populations in mid-2019 ranging from 252 to 3784, with a mean of 783. Datazones comprising 20% of the population experiencing the highest rates of income deprivation were placed in Quintile 1 (most deprived) and the datazones which make up the 20% of the population experiencing lowest rates of income deprivation were placed in Quintile 5 (least deprived).

### 2.4 Age-standardised mortality rates

All-cause and cause-specific age-standardised mortality rates (ASMRs) were calculated for each year separately using the 2013 European Standard Population (Eurostat, 2013), and grouping the data into 5-year age bands 0–4, 5–9, 10–14, …, 85–89 and 90+. Findings are presented separately for premature mortality (ages 0–74) and for deaths of those aged 75+ years.

### 2.5 Life tables

Multiple decrement life tables were constructed for the calculation of diversity in causes of mortality for Scotland as a whole, and for each SIMD income deprivation quintile. Life tables for single year age groups were calculated using established methods for each year from 2001 to 2019 (Veron et al., 2002). Rates of mortality were calculated from NRS mortality and population data from ages 0 to 89. Using a single open-ended age category from 90+ was considered undesirable because of the substantial number of deaths which occur in this age group. Mortality rates were therefore extrapolated for ages 90–110+ for each year and sex for Scotland as a whole and for each SIMD income deprivation quintile. A Kannisto-Makeham (Vaupel et al., 1998) logistic model was used for these extrapolations using version 1.9.3 of the package *MortalityLaws* on R version 4.0.2 (Pascariu, 2022; R Core Team, 2020).

### 2.6 Diversity

In this study, we measure normalised alpha diversity in causes of mortality using death counts extracted from multiple-decrement life tables. Diversity was calculated as described in the Reeve et al. (2016) framework for the measurement of diversity. Building on the work of Hill (Hill, 1973), the viewpoint parameter, *q*, is varied within its range of 0 to infinity to determine the weight attributed to relative abundance (prevalence). Normalised alpha diversity at q = 1, as used in this study, weights each type (cause of mortality) exactly in proportion to its prevalence, treating all individuals equally. It is an ‘effective number’ of causes of mortality formulation of Shannon entropy which describes the average uncertainty associated with predicting the cause of mortality of a single mortality record picked randomly. It captures information about both the number of causes of mortality in the sample and their prevalence, in which each type (cause) is weighted exactly by its prevalence. Calculating diversity in this way can give rise to values between 1 (if all deaths are attributed to one cause of mortality) and the total number of causes of mortality present (if all causes of mortality are equally prevalent); a higher value of diversity therefore indicates a more even distribution. Diversity can be thought of as measuring as the effective number of causes in each population; this is the number of equally prevalent causes needed to produce a group with equivalent diversity (Jost, 2007). Using an effective number of causes in this way, we can easily compare between populations and interpret differences in diversity. Calculations of diversity were performed using version 2.0 of the *rdiversity* (Mitchell et al., 2020) package, on R version 4.0.2 (R Core Team, 2020).

### 2.7 Analyses

To analyse and compare death rates between populations, ASMRs, calculated in each year from 2001 to 2019, separately for men and women, and distinguishing between those younger than 75 years (premature mortality) and for those aged 75 years and older.

To analyse diversity in causes of mortality, the normalised alpha diversity at *q* = 1 of ICD-10 three-character codes was calculated using multiple-decrement life tables (see Section 2.5) separately for those aged 0–74 and 75+ years. In the calculation of diversity, each group – such as subpopulations in a certain year – is known as a subcommunity; in this case consisting of the mortality records in each year for males and females separately. These age groups were chosen for two reasons. Firstly, the study of premature mortality is a longstanding priority in Scotland where rates of mortality for those amongst in this group have lagged improvements in comparable countries (Fenton et al., 2019). Secondly, the calculation of diversity can be heavily biased by the number of deaths within a population and further age breakdowns, especially when also considering the SIMD income deprivation quintiles separately, may lead to unreliable diversity calculations. For comparison, Supplementary Fig. 2 shows diversity in causes of mortality in males and females in deaths by twenty-year age groups.

ASMR and the normalised alpha diversity of mortality causes were calculated within each SIMD deprivation quintile, separately for premature mortality and deaths in those aged 75+ years. In the calculation of diversity, subcommunities consisted of the mortality records in each year in each deprivation quintile.

## 3 Results

### 3.1 Overall trends

Across Scotland, we find consistent trends from 2001 to 2019 of increasing diversity in causes of mortality in both men and women, alongside reductions in ASMRs. ASMRs in men younger than 75 years (premature mortality) fell from 749 per 100,000 population in 2001 to 497 per 100,000 population in 2019 (34% reduction), while women under 75 years experienced a smaller reduction from 440 per 100,000 population to 335 (24% reduction). The rate of deaths among those aged 75+ years fell for men from 11,453 per 100,000 population in 2001 to 8,836 in 2019 (23% reduction) with, again, a slightly smaller reduction for women from 8,679 per 100,000 population to 7,229 (17% reduction).

**Fig. 1:**
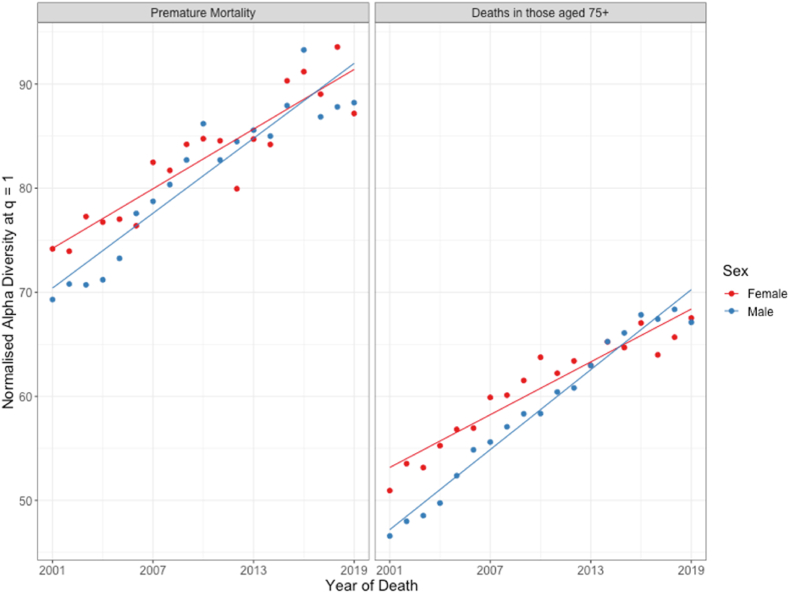
The trend in normalised alpha diversity at q = 1 of causes of mortality in males and females from 2001 to 2019, for premature mortality (deaths among those younger than 75 years) and for deaths amongst those older than 75 years. Each point represents the normalised alpha diversity at q = 1 of causes of mortality in the population at the level of ICD-10 three-character codes, plotted against the year of death. Solid lines represent linear regression across the years 2001–2019.

Fig. 1 shows trends in diversity of the causes of mortality over time by sex, distinguishing between premature mortality and deaths among those 75 and older. In this study diversity is calculated from distributions of mortality causes extracted from multiple decrement life tables to avoid biases arising from differences in population structure. However, trends in diversity in mortality cases measured from observed distributions of mortality causes are found to be qualitatively similar. Diversity was higher for those in the premature mortality group than for those in the older age group. Increases in the diversity of mortality causes occurred at similar rates in both premature and 75+ years mortality. Initially, diversity was higher for females than for males, but converged to similar values for the two sexes over the period. Analysis using more fine-grained age groups shows trends are comparable, except for deaths among those aged less than 40 years, where the number of deaths is relatively small and thus trends are likely to be less reliable (see also Supplementary Fig. 2 and associated discussion). Increasing age at death has previously been suggested as a driver of increasing diversity in mortality causes (Bergeron-Boucher et al., 2020). Here we find increases in age at death across the total population from 2001 to 2019, such that increases in age at death could be a contributing factor to increasing diversity in mortality causes. However, this increase in age at death across the population is driven by changes in the 75+ age group, with very little difference observed for the younger age group, so any effect of a change in age at death on diversification is likely to apply only to the older age group (Supplementary Table 1).

**Fig. 2:**
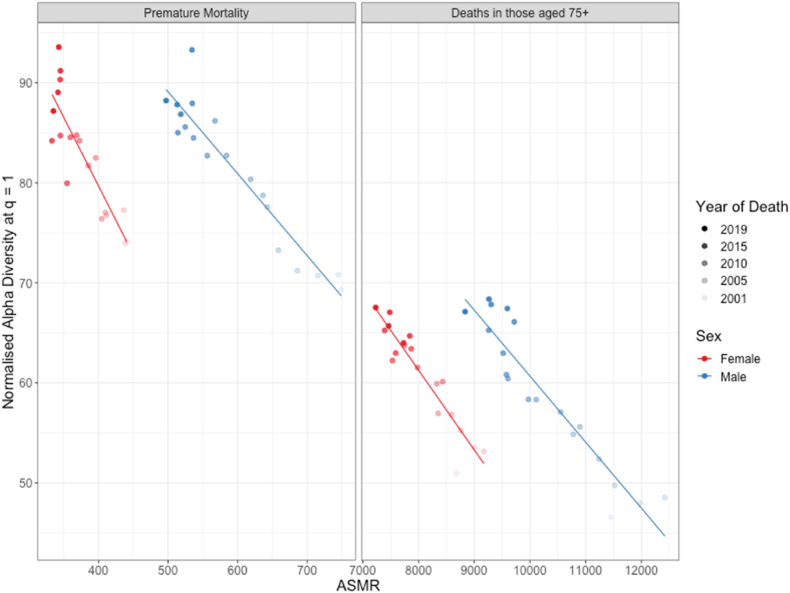
The relationship between the diversity in causes of mortality and age standardised mortality rate in Scotland in the years 2001–2019 in females and males; for premature mortality (deaths among those younger than 75 years) and for deaths amongst those older than 75 years. Each point represents the normalised alpha diversity at q = 1 of causes of mortality in the population at the level of ICD-10 three-character codes, plotted against the age-standardised mortality rate (per 100,000) of the population in a given year. Solid lines represent linear regression..

Fig. 2 shows the relationship between diversity in causes of mortality and age-standardised mortality rate, demonstrating that lower mortality rates were associated with higher diversity in causes. This finding shows that higher diversity is not simply driven by additional deaths. The increase in diversity of mortality causes despite the fall in mortality rates suggests that deaths associated with the leading mortality causes have reduced faster than the remaining causes. This leads to the distribution of causes becoming more even and therefore more diverse under this measure of diversity. This is borne out in the data: the five mortality causes with the highest ASMR in 2001 among those 75 years and older contributed to ∼40% of the all-cause ASMR in both men and women (causes listed in Supplementary Table 2); the combined mortality rate of these causes fell 58% in women and 49% in men from 2001 to 2019 whereas the mortality rate of all other causes fell only 10% in men and increased by 5% in women (Supplementary Table 3).

Men faced higher mortality rates than women throughout the period 2001 to 2019 (Supplementary Table 4). Our result show that, in general, higher mortality rates are associated with lower diversity in mortality causes, so we would also have expected diversity in causes of mortality among men to be lower than among women. However, Fig. 1 shows that men faced more diverse causes of mortality than women towards the end of the study period, demonstrating that this association between death rates and diversity does not hold between sexes.

### 3.2 Trends by deprivation

In Scotland, both men and women face considerably higher rates of death in more deprived areas; the percentage difference in ASMR between the most and least deprived fifth of the population has increased from 2001 to 2019, in both sexes and in both premature mortality and deaths in those 75 years and older. In fact, in 2019, both men and women younger than 75 years in the most deprived population fifth faced mortality rates more than 100% higher than those in the least deprived fifth of the population (Supplementary Table 5).

**Fig. 3:**
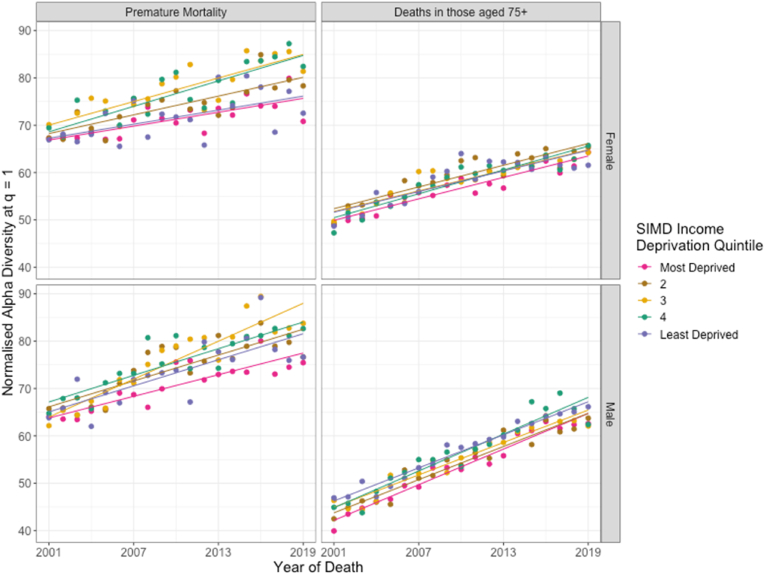
The trend in normalised alpha diversity at q = 1 of causes of mortality across SIMD Income Deprivation Quintiles in males and females from 2001 to 2019, for premature mortality (deaths among those younger than 75 years) and for deaths amongst those older than 75 years. Each point represents the normalised alpha diversity at q = 1 of causes of mortality in the population at the level of ICD-10 three-character codes, plotted against the year of death. Solid lines represent linear regression across the years 2001–2019.

Fig. 3 shows the trends over time in diversity, similarly to Fig. 1, broken down by SIMD income deprivation quintile. Across both sexes, we observe an increase in the diversity of mortality causes from 2001 to 2019 in both premature mortality and deaths among those 75+ years in all deprivation quintiles. In general, we observe a tendency for more diverse sets of mortality causes to be found among less deprived communities. We see this pattern most strongly in deaths in men aged 75 years and older. However, it can be seen in Fig. 3 that exceptions to this tendency are common; in most cases, diversity in mortality causes does not neatly increase from most to least deprived. That is, compared to the persistently patterned inequalities in mortality rate between deprivation quintiles, the diversity in mortality causes is not systematically ordered by area-level deprivation in the same way.

Furthermore, the rate at which diversity in mortality causes increased is not clearly patterned by deprivation. Mortality rates fell fastest in the most deprived areas; however, diversity did not increase most quickly in the areas where the fastest improvements in mortality rates occurred. Diversity increased at similar rates across quintiles in deaths among those aged 75+ years and although trends are more varied for premature mortality, they are not patterned by deprivation.

**Fig. 4:**
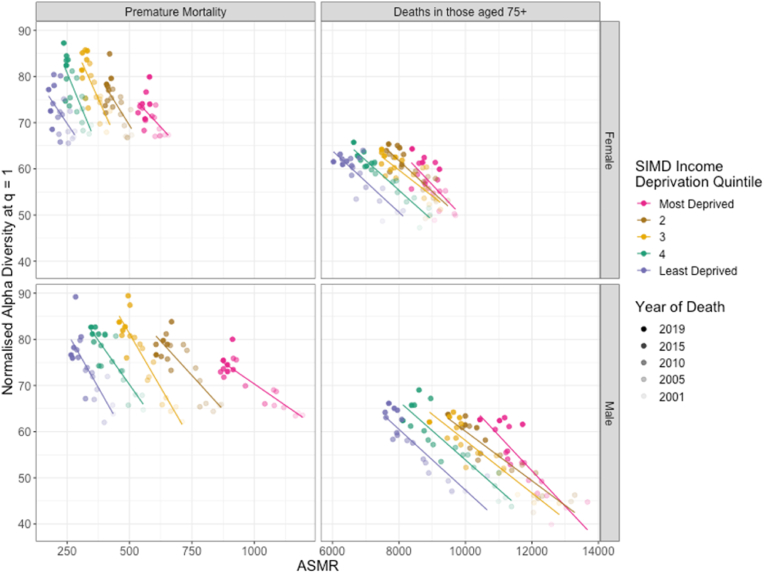
The relationship between the diversity of mortality causes and standardised mortality rate across SIMD Income Deprivation Quintiles in the years 2001–2019 in females and males; for premature mortality (deaths among those younger than 75 years) and for deaths amongst those older than 75 years. Each point represents the normalised alpha diversity at q = 1 of causes of mortality in the population at the level of ICD-10 three-character codes plotted against the age-standardised mortality rate (per 100,000) of the population in a given year. Solid lines show the linear regression of diversity on ASMR.

Fig. 4 shows the relationship between the mortality rate and the diversity of mortality causes, broken down by deprivation quintile. The pattern is largely similar across SIMD income deprivation quintiles, in both age groups and sexes, with increases in diversity in mortality causes negatively related with mortality rates, which fell over time period. Further, it can be seen in Fig. 4 that for a given ASMR, comparison between deprivation quintiles and years shows that different levels of diversity can occur. Different subpopulations therefore achieve given levels of ASMR at different levels of diversity, suggesting that different sectors of the population are achieving improvements in death rates in distinct ways, through different shifts in the distribution of mortality causes.

## 4 Discussion

We find that, in Scotland, reductions in age-standardised mortality rates have been accompanied by increases in the diversity of mortality causes, both for premature mortality and deaths among those aged 75+ years. Falling mortality rates and increasing diversity in the causes of mortality within Scotland indicates that mortality associated with the most common causes of death is reducing at a faster rate than other causes. This drives the reduction in mortality rates as a whole and leads to a reduction in the proportion of deaths caused by these most common causes, creating a more even distribution. This is likely to be partially as a result of public health success. For example, reducing the prevalence of heart attacks and strokes has been a priority of the Scottish Government for many years (Scottish Government, 2014) because relative rates of these diseases in Scotland are higher than comparable areas of the rest of the UK and Europe (Mitchell et al., 2005) and the resultant decrease may have had the effect of creating a more even and diverse distribution of mortality causes. Treatment for heart attacks and strokes and ongoing management of related conditions, as well as cancer treatment, have also improved significantly over the period (Critchley et al., 2003). In contrast, a number of causes of mortality have become more prevalent despite public health measures, with mortality rates increasing during the study period. Most notably among these are deaths of despair which primarily affect younger people and those in more deprived areas, and degenerative diseases which primarily affect the elderly (Allik et al., 2020; Ramsay et al., 2020). In the respective affected subpopulations these increases can lead to a more even distribution of mortality causes because once rare causes constitute an increasing proportion of deaths.

Previous studies have analysed differences in diversity in mortality causes across countries (Bergeron-Boucher et al., 2020; Izsak, 1993), and the only previous comparison of subpopulations within countries has been between sexes. In most countries, Bergeron-Boucher et al. (2020) find higher diversity in mortality causes among women. Direct comparison of diversity in causes of mortality between men and women is complicated by the biological differences in possible causes of mortality between sexes. Our findings do, however, show diversity in causes of mortality has increased more quickly among men than women. We note a similar relationship between ASMRs and diversity in mortality causes in both sexes and more rapid diversification in men may be explained by the larger improvements noted in mortality rates. More rapid diversification in men may be due to the larger decrease in cardiovascular disease as the dominant cause of mortality and the more rapid increases in a number of causes of mortality such as deaths of despair which are linked to behavioural risk factors (Augarde et al., 2022; Brown et al., 2019).

Our findings offer an insight into how diversity in mortality causes varies across subpopulations within Scotland at a level not previously studied. We show that for the most part, among men in Scotland, a higher level of economic deprivation is associated with a more diverse set of mortality causes, although we observe exceptions to this, and variation in recent trends in the diversity of causes among men have caused this relationship to weaken over time. We find little evidence of any association between deprivation and diversity in mortality causes in women. This difference may be partially explained by evidence that in recent years, relative inequalities between socioeconomic groups in all-cause, and in some cases cause-specific mortality, are smaller among women than men (Brown et al., 2019).

We show that the trend of increasing diversity in mortality causes as mortality rates fall holds across income deprivation quintiles. Our results also show that despite increasing inequalities in mortality rates between the most and least income deprived quintiles of the population and the clear pattern linking mortality rates with diversity of mortality causes, the diversity of mortality causes is not clearly correlated with deprivation. This suggests that inequalities in mortality rates do not fully explain observed differences in the diversity of causes between quintiles of deprivation.

The differing relationship between ASMRs and diversity in causes of mortality highlights that though mortality rates have improved across the country, this is not a uniform transition from high to low mortality rates. Rather, sections of the population are achieving these improvements in distinct ways, altering the distribution of mortality causes differently.

Previously, it has been implied that diversification in the causes of mortality across populations is driven by the aging of the population (Bergeron-Boucher et al., 2020), with evidence suggesting that those who reach older ages are susceptible to a wider range of ailments, increasing the diversity of their causes of death. It is possible that this hypothesis explains some of the increase in diversity we observe given that life expectancy has also increased across Scotland from 2001 to 2019 (National Records of Scotland, 2021). However, as we note, diversity in mortality causes is not clearly correlated with area-level deprivation despite life expectancy being consistently higher in less deprived areas. This suggests that increasing age at death does not fully explain the observed diversification in mortality causes or differences between subpopulations in Scotland.

We also find that in each year studied, a more diverse set of mortality causes occurred in for premature mortality than deaths among those aged 75+ years. A possible explanation for this is the inclusion of infant deaths and deaths among the very young in premature mortality. Many of the causes of mortality in the very young do not appear among adults and therefore can be expected to increase the diversity of the group. However, in additional analysis with deaths in those younger than 15 years excluded from the premature mortality age group, the diversity of mortality causes among those aged 15 to 74 remained higher than among all younger than 75 years, precluding this explanation (Supplementary Fig. 3).

This study examines mortality in Scotland in the period directly preceding the COVID-19 pandemic. The burden of mortality has changed during the pandemic both through the introduction of this novel cause of mortality and through various interactions with other mortality causes and risk factors. Further study of diversity in mortality causes should examine how the pandemic has affected trends and how the study of diversity in mortality can improve the understanding of the effect of the pandemic on causes of mortality not directly related to COVID-19.

### 4.1 Strengths and limitations

Using the diversity of causes of mortality at the level of individual ICD-10 three-character codes in population health relies on highly accurate mortality data; in this study, we utilise underlying cause of death data extracted from NRS mortality records. These government statistics are the best available data on mortality in Scotland; however, concerns have been raised internationally about the reliability of cause of death information (Alpérovitch et al., 2009; Zellweger et al., 2019). Socioeconomic conditions have been found to influence the reliability of data on causes (Zellweger et al., 2019) and some of the variation noted across income deprivation quintiles may be attributable to these errors. The Scottish Government’s Living and Dying Well action plan, introduced in 2008, led to an increase in the proportion of the population dying and spending their last 6 months at home or in a community setting (relative to hospital), which may also increase uncertainty in the recorded primary cause of mortality (NHS Scotland Information Services Division, 2018). Further to these sources of uncertainty, an increasing proportion of deaths in Scotland have been attributed to ill-defined causes (Mesalles-Naranjo et al., 2018). While the uncertainty surrounding the accuracy of codes is likely to be small across the total population, it is unlikely to be possible to eliminate it or to find a more reliable source of data for the calculation of diversity.

During the period of this study, two significant updates in coding practices were made by National Records of Scotland, occurring in 2010 (National Records of Scotland, 2011) and 2016 (National Records of Scotland & Scottish Government, 2017). The largest effects of the first were the consolidation of deaths of despair – deaths attributed to suicide or associated with alcohol or drugs (Allik et al., 2020) – within the ICD-10 chapter *External causes of morbidity and mortality* and of certain degenerative diseases under ICD-10 chapter *Diseases of the Nervous System,* increasing the number of deaths recorded within each chapter. In Fig. 3, Fig. 4 we can see the effect of these updates as changes in the trends in diversity and mortality rates. The second update had smaller effects, with the most notable an increase in the number of deaths classified as Dementia, which is within *Diseases of the Nervous System,* and a corresponding decrease in the number of deaths coded under respiratory diseases*.* While the first of these has a noticeable effect on the diversity and mortality rate within several chapters, neither is likely to affect results to a significant degree.

Many other measures of alpha diversity are available within the literature – and indeed within the framework used in this study – and could be applied to mortality data. Normalised alpha diversity at q = 1 (equivalent to Shannon entropy) is used here because it takes into account the relative prevalence of causes, unlike less conservative measures such as species richness, while avoiding the effective exclusion of rare causes in more conservative measures (Reeve et al., 2016). Further, the measures used here measure diversity in an effective number of types formulation, allowing straightforward interpretation and comparison between populations.

## 5 Conclusions

We find increasing diversity in mortality causes as overall mortality rates fall across subpopulations in Scotland. This suggests reductions in mortality have been driven by reductions in the most common causes of mortality and that a redistribution of deaths to other causes has occurred creating a more even distribution.

We note that while men have historically faced a less diverse set of mortality causes, they have seen greater diversification of causes in recent years. This has come alongside reducing inequalities in mortality rates but has occurred at a much faster rate.

The results of this study show that the diversity of mortality causes is increasing across all deprivation quintiles of the Scottish population; however, the rate of increase varies between quintiles and we do not find evidence that this variation is patterned by deprivation. Given the close link between deprivation and mortality rates, this means that across deprivation quintiles the lowest mortality rate has not always been associated with the most diverse set of mortality causes.

The observed increases in diversity of mortality causes across sexes, deprivation quintiles and age groups is a sign that a more holistic approach to the improvement of health in low mortality countries is increasingly necessary. More emphasis is likely needed on reducing the rate of deaths from uncommon causes where funding may currently be limited. This presents a multifaceted problem for public health professionals and the health care sector, for which the diversity of mortality causes can be a gauge.

## Ethics approval

We use secondary data for which ethics approval was not required.

## Funding

CM’s contribution to this work was funded by the 10.13039/501100000265Medical Research Council (MC_UU_00022/4) and the 10.13039/100012095Scottish Government 10.13039/501100000589Chief Scientist Office (SPHSU19) and under a 10.13039/501100000265Medical Research Council and 10.13039/501100000853University of Glasgow College of Medical, Veterinary and Life Sciences PhD studentship (MC_ST_U18004). DB’s contribution to this work was funded by the 10.13039/501100000265Medical Research Council (MC_UU_00022/2) and the 10.13039/100012095Scottish Government 10.13039/501100000589Chief Scientist Office (SPHSU17). RR’s contribution to this work was funded by 10.13039/501100000271Science and Technology Facilities Council (grant number ST/V006126/1), 10.13039/501100000268Biotechnology and Biological Sciences Research Council (BB/R012679/1), and 10.13039/501100000270Natural Environment Research Council (grant numbers NE/T004193/1 and NE/T010355/1). RM’s contribution to this work was supported by The Leckie Fellowship, 10.13039/501100000265Medical Research Council (grant number MC_UU_00022/4) and the 10.13039/501100000589Chief Scientist Office (grant number SPHSU19).

## Author contributions

**Ciaran McMonagle**: Conceptualisation; Methodology; Software; Formal analysis; Investigation; Data curation; Writing - Original draft; Visualisation. **Denise Brown**: Resources; Writing – Review and Editing; Supervision. **Richard Reeve**: Writing – Review and Editing; Supervision. **Rebecca Mancy**: Conceptualisation; Writing – Review and Editing; Supervision.

## Declaration of competing interest

None.

## Data Availability

The authors do not have permission to share data.
