# Supplementary material for: Trends in the diversity of mortality causes and age-standardised mortality rates among subpopulations within Scotland, 2001–2019
Source: SSM Popul Health. 2022 Aug 13;19:101192. doi: 10.1016/j.ssmph.2022.101192 (PMC9418986; doi:10.1016/j.ssmph.2022.101192)
Supplement: Multimedia component 1 [file mmc1.docx]

**Supplementary Materials**

Supplementary Figure 1 shows normalised alpha diversity at q=0 in causes of mortality. This measure weights causes of mortality equally and is therefore equivalent to a count of the number of mortality causes in each year.

**
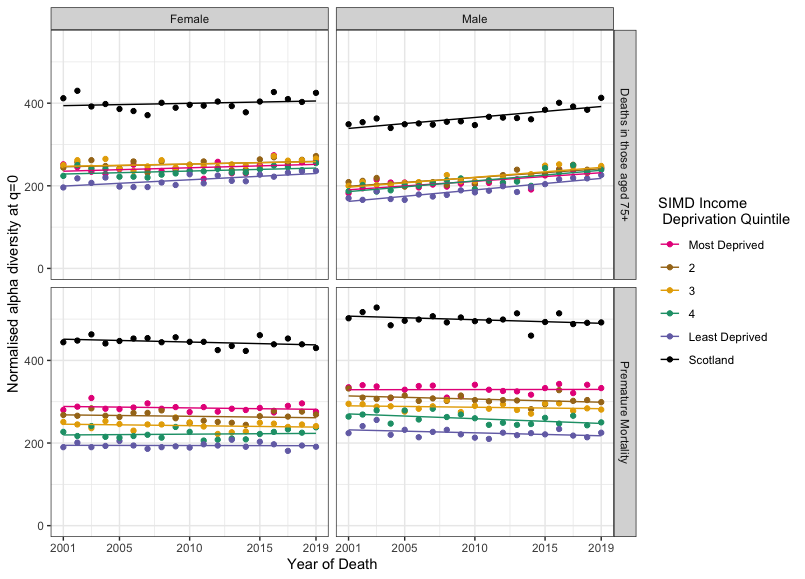
**

Supplementary Figure 1: The trend in normalised alpha diversity at q=0 of causes of mortality (equivalent to the number of recorded causes) across SIMD Income Deprivation Quintiles in males and females from 2001-2019, for premature mortality (deaths among those younger than 75 years) and for deaths amongst those older than 75 years. Each point represents the normalised alpha diversity at q=0 of causes of mortality in the population at the level of ICD-10 three-character codes, plotted against the year of death. Solid lines represent linear regression across the years 2001-2019

Supplementary Figure 2 shows normalised alpha diversity in causes of mortality for the males and females in the Scottish population in deaths within twenty-year age groups. Under this more fine-grained age breakdown differences in trends in diversity in mortality causes are shown. In those aged 40+ there are increases in diversity in mortality causes, whereas below age 40, causes of mortality became less diverse over the period. Results for the younger groups should be treated carefully, because lower rates of mortality give rise to greater stochastic variation in diversity. (Deaths in the age groups 0 to 19 years and 20 to 39 years represent <1% and <2% of total deaths in each year respectively). Reductions in diversity at these ages may be linked to the rising prevalence of deaths of despair among younger people and especially young men in Scotland. Deaths of despair are increasingly dominant in these age groups causing a reduction in diversity. Across the premature mortality age range as a whole other causes dominate the distribution of mortality meaning the increasing prevalence of deaths of despair causes a more even distribution.


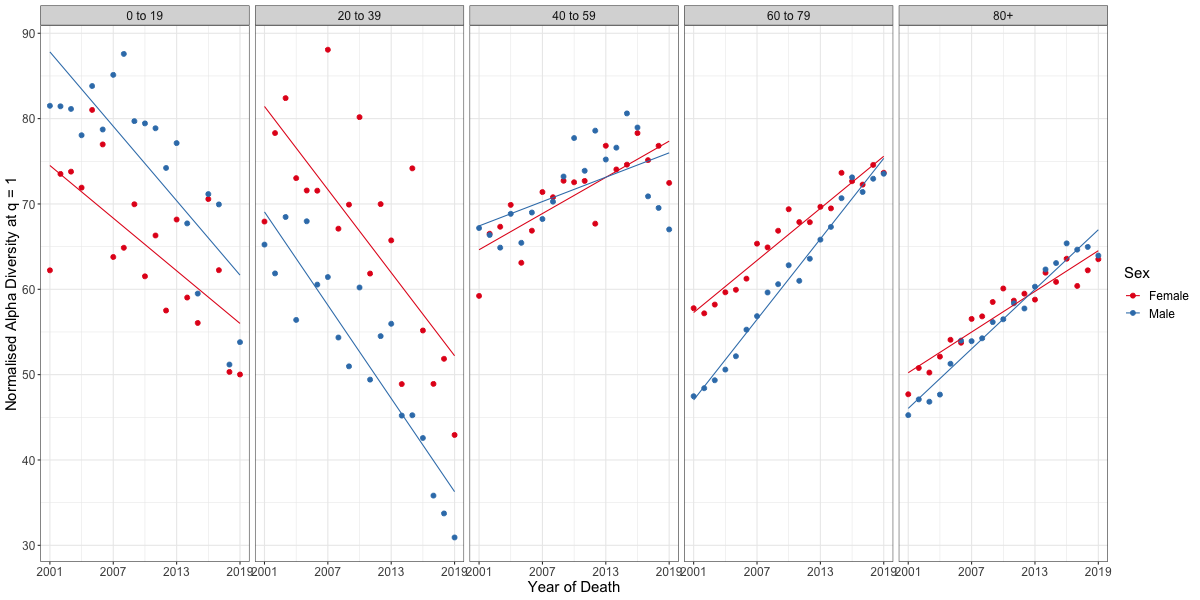


Supplementary Figure 2: The trend in normalised alpha diversity at q=1 of causes of mortality in males and females from 2001-2019, for deaths among those aged 0 to 19 years, 20 to 39 years, 40 to 59 years, 60 to 79 years and 80+ years. Each point represents the normalised alpha diversity at q=1 of causes of mortality in the population at the level of ICD-10 three-character codes, plotted against the year of death. Solid lines represent linear regression across the years 2001-2019.

Supplementary Table 1: Median age at death and standard deviation in the distribution of age at death from 2001-2019 across Scotland in males and females across deaths at all ages, premature mortality, and deaths in those aged 75 and older.

|  | **SIMD Quintile** | **Median age at death in 2001** | **Standard deviation of age at death in 2001** | **Median age at death in 2019** | **Standard deviation of age at death in 2019** |
| --- | --- | --- | --- | --- | --- |
| **All ages** |  |  |  |  |  |
| **Female** |  |  |  |  |  |
|  | 1 | 78 | 15.3 | 79 | 15.8 |
|  | 2 | 79 | 13.9 | 81 | 14.5 |
|  | 3 | 81 | 13.8 | 83 | 13.4 |
|  | 4 | 82 | 14.3 | 84 | 14.0 |
|  | 5 | 82 | 15.5 | 84 | 13.0 |
|  | Whole Country | 80 | 14.6 | 82 | 14.4 |
| **Male** |  |  |  |  |  |
|  | 1 | 70 | 16.7 | 72 | 17.0 |
|  | 2 | 73 | 15.7 | 76 | 16.0 |
|  | 3 | 75 | 15.2 | 78 | 14.5 |
|  | 4 | 76 | 15.8 | 79 | 14.3 |
|  | 5 | 75 | 15.6 | 80 | 14.0 |
|  | Whole Country | 74 | 16.0 | 77 | 15.7 |
| **Premature Mortality** | |  |  |  |  |
| **Female** |  |  |  |  |  |
|  | 1 | 65 | 14.1 | 63 | 13.5 |
|  | 2 | 66 | 13.0 | 65 | 12.7 |
|  | 3 | 66 | 13.4 | 66 | 11.8 |
|  | 4 | 65 | 14.1 | 65 | 12.7 |
|  | 5 | 65 | 15.3 | 67 | 11.8 |
|  | Whole Country | 66 | 13.8 | 65 | 12.7 |
| **Male** |  |  |  |  |  |
|  | 1 | 62.5 | 14.9 | 61 | 14.3 |
|  | 2 | 64 | 14.2 | 64 | 14.1 |
|  | 3 | 65 | 13.9 | 65 | 12.6 |
|  | 4 | 65 | 14.8 | 66 | 12.8 |
|  | 5 | 64 | 14.4 | 66 | 13.3 |
|  | Whole Country | 64 | 14.5 | 64 | 13.7 |
| **Deaths among those aged 75+** | | |  |  |  |
| **Female** | | |  |  |  |
|  | 1 | 84 | 6.1 | 85 | 6.4 |
|  | 2 | 84 | 6.1 | 86 | 6.3 |
|  | 3 | 85 | 6.2 | 87 | 6.3 |
|  | 4 | 86 | 6.3 | 87 | 6.3 |
|  | 5 | 86 | 6.4 | 87 | 6.4 |
|  | Whole Country | 85 | 6.2 | 86 | 6.4 |
| **Males** |  |  |  |  |  |
|  | 1 | 81 | 5.1 | 83 | 5.5 |
|  | 2 | 81 | 5.3 | 83 | 5.6 |
|  | 3 | 82 | 5.4 | 84 | 5.8 |
|  | 4 | 83 | 5.7 | 84 | 6.0 |
|  | 5 | 82 | 5.3 | 85 | 5.9 |
|  | Whole Country | 81 | 5.4 | 84 | 5.8 |

Supplementary Table 2: The ICD-10 codes and associated mortality causes most commonly assigned as the underlying cause of death in men and women in premature mortality and deaths among those aged 75+ in 2001 and 2019 and the percentage of deaths in that year associated with that cause.

| **ICD-10 Code** | **Associated Cause** | **Percentage (%) of deaths** |
| --- | --- | --- |
| **2001** |  |  |
| **Female** |  |  |
| **Premature Mortality** | |  |
| C34 | Malignant neoplasm of bronchus and lung | 9.7 |
| I21 | Acute myocardial infarction | 9.1 |
| C50 | Malignant neoplasm of breast | 7.0 |
| I25 | Chronic ischaemic heart disease | 5.9 |
| J44 | Other chronic obstructive pulmonary disease | 5.2 |
| **Deaths in those aged 75+** | |  |
| I21 | Acute myocardial infarction | 12.6 |
| I64 | Stroke, not specified as haemorrhage or infarction | 8.1 |
| I25 | Chronic ischaemic heart disease | 7.6 |
| J18 | Pneumonia, organism unspecified | 6.4 |
| F03 | Unspecified dementia | 5.1 |
| **Male** |  |  |
| **Premature Mortality** | |  |
| I21 | Acute myocardial infarction | 13.4 |
| C34 | Malignant neoplasm of bronchus and lung | 9.9 |
| I25 | Chronic ischaemic heart disease | 8.6 |
| K70 | Alcoholic liver disease | 4.0 |
| J44 | Other chronic obstructive pulmonary disease | 3.7 |
| **Deaths in those aged 75+** | |  |
| I21 | Acute myocardial infarction | 14.7 |
| I25 | Chronic ischaemic heart disease | 8.4 |
| C34 | Malignant neoplasm of bronchus and lung | 6.6 |
| J44 | Other chronic obstructive pulmonary disease | 6.0 |
| I64 | Stroke, not specified as haemorrhage or infarction | 5.4 |
| **ICD-10 Code** | **Associated Cause** | **Percentage (%) of deaths** |
| **2019** |  |  |
| **Female** |  |  |
| **Premature Mortality** | |  |
| C34 | Malignant neoplasm of bronchus and lung | 11.5 |
| J44 | Other chronic obstructive pulmonary disease | 7.0 |
| C50 | Malignant neoplasm of breast | 5.9 |
| I21 | Acute myocardial infarction | 4.6 |
| X42 | Accidental poisoning by and exposure to narcotics and psychodysleptics [hallucinogens], not elsewhere classified | 3.0 |
| **Deaths in those aged 75+** | |  |
| G30 | Alzheimer disease | 7.8 |
| F01 | Vascular dementia | 6.0 |
| F03 | Unspecified dementia | 5.7 |
| I21 | Acute myocardial infarction | 5.7 |
| C34 | Malignant neoplasm of bronchus and lung | 4.9 |
| **Male** |  |  |
| **Premature Mortality** | |  |
| C34 | Malignant neoplasm of bronchus and lung | 8.8 |
| I21 | Acute myocardial infarction | 8.4 |
| I25 | Chronic ischaemic heart disease | 5.8 |
| X42 | Accidental poisoning by and exposure to narcotics and psychodysleptics [hallucinogens], not elsewhere classified | 5.5 |
| J44 | Other chronic obstructive pulmonary disease | 4.0 |
| **Deaths in those aged 75+** | |  |
| I21 | Acute myocardial infarction | 8.5 |
| C34 | Malignant neoplasm of bronchus and lung | 5.9 |
| F01 | Vascular dementia | 4.9 |
| I25 | Chronic ischaemic heart disease | 4.8 |
| J44 | Other chronic obstructive pulmonary disease | 4.8 |

| Supplementary Table 3: Percentage change from 2001 to 2019 in the age-standardised mortality rate of: the five most common mortality causes in 2001 and across all causes in 2001 in Scotland in males and females aged 75 and older and premature mortality. | **Percentage (%) change in age-standardised mortality rate from 2001-2019** | |
| --- | --- | --- |
|  | **The five most common causes of mortality in 2001** | **All other causes of mortality** |
| **Females** | | |
| Premature Mortality | -38 | -27 |
| Deaths in those aged 75+ | -58 | 5 |
| **Males** | | |
| Premature Mortality | -52 | -27 |
| Deaths in those aged 75+ | -49 | -10 |

Supplementary Table 4: Percentage difference in age-standardised mortality rate and the normalised alpha diversity at q=1 of mortality causes between males and females in Scotland

In each year from 2001 to 2019 in premature mortality and deaths among those aged 75+.

|  | **Percentage (%) difference between males and females** | | | | | | | | | | | | | | | | | | |
| --- | --- | --- | --- | --- | --- | --- | --- | --- | --- | --- | --- | --- | --- | --- | --- | --- | --- | --- | --- |
|  | 2001 | 2002 | 2003 | 2004 | 2005 | 2006 | 2007 | 2008 | 2009 | 2010 | 2011 | 2012 | 2013 | 2014 | 2015 | 2016 | 2017 | 2018 | 2019 |
| **Premature Mortality** |  |  |  |  |  |  |  |  |  |  |  |  |  |  |  |  |  |  |  |
| Diversity in Mortality Causes | -6.8 | -4.3 | -8.9 | -7.5 | -5.0 | 1.5 | -4.7 | -1.7 | -1.8 | 1.7 | -2.2 | 5.5 | 1.0 | 0.9 | -2.7 | 2.3 | -2.5 | -6.3 | 1.2 |
| Age-Standardised Mortality Rate | 51.9 | 51.8 | 48.5 | 50.0 | 46.6 | 45.4 | 46.6 | 46.5 | 44.2 | 42.5 | 43.0 | 40.8 | 41.3 | 42.8 | 43.2 | 43.0 | 41.1 | 39.8 | 39.0 |
| **Deaths in those aged 75+** |  |  |  |  |  |  |  |  |  |  |  |  |  |  |  |  |  |  |  |
| Diversity in Mortality Causes | -9.0 | -10.9 | -9.1 | -10.5 | -8.2 | -3.7 | -7.4 | -5.2 | -5.3 | -8.9 | -2.9 | -4.2 | 0.0 | 0.0 | 2.1 | 1.2 | 5.2 | 4.0 | -0.6 |
| Age-Standardised Mortality Rate | 27.5 | 28.4 | 30.1 | 27.3 | 26.7 | 25.4 | 26.7 | 22.3 | 23.6 | 25.1 | 24.2 | 19.6 | 22.6 | 22.4 | 21.4 | 21.7 | 21.5 | 21.5 | 20.0 |

Supplementary Table 5: Percentage difference in ASMR between the least and most income deprived quintiles in Scotland in 2001 and 2019 in men and women in premature mortality and deaths among those aged 75+

|  | **Percentage (%) difference in ASMR between most and least deprived population fifths** | |
| --- | --- | --- |
|  | **2001** | **2019** |
| **Females** |  |  |
| Premature Mortality | 77.3 | 101.3 |
| Deaths in those aged 75+ | 18.5 | 32.1 |
| **Males** |  |  |
| Premature Mortality | 91.9 | 106.7 |
| Deaths in those aged 75+ | 26.6 | 30.0 |

Supplementary Table 6: Percentage change from 2001-2019 in the diversity of mortality causes and age-standardised mortality rates in premature mortality and deaths among those aged 75+ in men and women across SIMD deprivation quintiles in Scotland.

|  |  |  | **Percentage (%) change 2001 to 2019** | | |  |
| --- | --- | --- | --- | --- | --- | --- |
|  |  | **Most deprived** | **2** | **3** | **4** | **Least Deprived** |
| **Premature Mortality** | |  |  |  |  |  |
| **Female** | Diversity in Mortality Causes | 5.3 | 16.4 | 16.1 | 18.7 | 8.4 |
|  | Age-Standardised Mortality Rate | -9.9 | -19.7 | -25.5 | -28.7 | -33.5 |
| **Male** | Diversity in Mortality Causes | 17.8 | 16.6 | 34.8 | 27.7 | 20.1 |
|  | Age-Standardised Mortality Rate | -25.5 | -29.5 | -35.5 | -37.7 | -38.9 |
| **Deaths in those aged 75+** | |  |  |  |  |  |
| **Female** | Diversity in Mortality Causes | 31.6 | 32.7 | 29.3 | 39.1 | 26.4 |
|  | Age-Standardised Mortality Rate | -7.4 | -12.6 | -16.0 | -23.0 | -19.7 |
| **Male** | Diversity in Mortality Causes | 56.9 | 50.0 | 33.9 | 38.9 | 41.0 |
|  | Age-Standardised Mortality Rate | -17.1 | -21.3 | -22.9 | -26.2 | -19.7 |
